# Editorial: Insights in avian physiology

**DOI:** 10.3389/fphys.2023.1235728

**Published:** 2023-06-23

**Authors:** C. G. Scanes, S. G. Velleman

**Affiliations:** ^1^ Department of Biological Science, University of Wisconsin Milwaukee, Milwaukee, WI, United States; ^2^ Department of Animal Sciences, The Ohio State University, Columbus, OH, United States

**Keywords:** genetics, reproduction, muscle, signal transduction, environment, nutrition

This Research Topic provides a compendium of eleven papers and reviews on avian physiology. Birds are successful in different climatic zones including polar, tropical, dry climates, moist subtropical, moist continental mid-Latitudes and highlands. It has been estimated that there are globally 18,043 avian species (95% confidence interval 15,845–20,470). This is in contrast to earlier estimates of 9,000–10,000 species (Barrowclough et al., 2016) and compares with 6,495 mammalian species (96 recently extinct, 6,399 extant) (Burgin et al., 2018). This Research Topic includes papers, opinion pieces and reviews on the physiology of birds.

Two papers cover the physiology of wild birds. In red-headed buntings subjected to simulated migration, there are shifts in reticulocytes (increased) and apoptosis of erythrocytes together with expression of stress oxidation related genes in blood (Bhardwaj et al.). Moreover, Kumar et al. review their and others studies on migration in Palearctic-Indian migratory buntings, the blackheaded bunting (*Emberiza melanocephala*) and redheaded bunting (Emberiza bruniceps).

Globally, there have been large increases in the production of poultry (chickens, turkeys, ducks and geese) and livestock for meat (Table 1: FAOSTAT, 2023). The development of poultry production can be broadly attributed to the following.

• Large genetic improvements,
• Nutrition to meet bird requirements,
• Vaccinations and health improvement,
• Efficient production systems.

**TABLE 1:** Shifts in global production of meats by species together with eggs between 1991 and 2021 (FAOSTAT, 2023).

Species	Global production in million metric tons	
	1991	2021
Meats		
Chicken	38.2	121.6
Pig	70.7	120.4
Cattle	54.2	74.4
Sheep	6.9	10.0
Goats	2.7	6.4
Ducks	1.4	6.2
Turkey	3.5	5.8
Geese	0.8	4.4
Eggs		
Chicken	38.2	86.4
Ducks and geese	3.05	6.17

Geneticists have been responsible for much of the large increases in the growth rate, particularly that of breast muscle, in young meat chickens or broilers (Siegel). They utilized the diverse storehouse of genes in different populations of chickens, the heritability of key traits and the short generation interval (Siegel). The shifts in growth rate and other production traits are based on the physiology of the poultry. There have been increases in the incidence and/or severity of myopathies (White striping, Wooden breast and Spaghetti breast) in broiler chickens (Bailey). There appears to be little genetic relationships between the myopathies and either growth or percentage breast muscle (Bailey). What is missing is more studies on reactive oxygen species and oxidative stress in the muscles and other organs in broiler type chickens.

Satellite cells are critical to muscle growth, itself the basis of meat production. Satellite cells are mono-nucleated stem cells that undergo asymmetric division generating two daughter cells:

• One playing a role in muscle growth and development.
• The other replenishing the satellite cell reservoir in the muscle.
• Satellite cells, as stem cells, can also develop into other cell types such as adipocytes (reviewed Velleman).

Velleman proposes that there are multiple populations of satellite cells. The corollary is that multiple populations of satellite cells have different properties and, therefore, functions. Among the different properties of such populations are the following:

• Growth potential,
• Signal transduction:
• Mechanistic target of rapamycin (mTOR) pathway,
• Wingless type mouse mammary tumor virus integration site family/planar cell polarity (Wnt/PCP) pathway,
• There are considerable implications to the presence of multiple forms of satellite cells including increase muscle growth and overcoming the problems of myopathies (reviewed Velleman).

Poultry provide a second food, namely, eggs (see Table 1). The Research Topic includes description of a new anterior pituitary hormone that appears related to reproduction. The pituitary gland expresses relaxin 3 (RLN3) in adult female but male chickens (Lv et al.). Expression of RLN3 *in vitro* by pituitary cells is increased by two releasing hormones, namely, gonadotropin releasing hormone and corticotropin releasing hormone and by estradiol (Lv et al.). Expression of two receptors for RLN3 have been examined in chickens with RXFP1 expressed in oviduct particularly infundibulum plus the brain but not the pituitary gland while RXFP3 is expressed in the kidneys, hypothalamus and spinal column but not the pituitary gland (Lv et al.). Lv et al. also propose that the avian RLN3 gene is a duplicated copy of the ancestral RLN3 gene.

Scanes reinterprets data from older studies on the timing of ovulation and oviposition in hens on ahemoral light cycles. He concludes that there is not a satisfactory model that fully accounts for the timing of ovulation/oviposition and now should be the time to develop one.

Environmental factors profoundly influence the physiology of birds. For instance, spectrum of light was demonstrated to influence reproduction in chickens with greater activation of the hypothalamo-pituitary axis in chickens photostimulated with red compared to green light (Rozenboim et al.). In addition, red but not green light increased expression of red opsin in the hypothalamus (Rozenboim et al.).

Reproductive development in female birds is accompanied by accumulation of calcium in bones. Bahry et al. examine the relationship between growth and nutrition using plasma concentrations of estradiol as an indicator of reproductive development together with bone mineralization.

There are other aspects of avian physiology included in the Research Topic. For example, Csillag et al. discuss the utility of chickens and songbirds as models for the typical failure symptoms associated with autism spectrum disorder (ASD). This intriguing concept provides a novel biomedical utilization of avian physiology. Pierzchala-Koziec and Scanes analyze the limited information on the neuropeptides and putative neuropeptides derived from three opioid genes, proenkephalin (PENK), prodynorphin (PDYN) and pronociceptin (PNOC). Data on the deduced structures of proenkephalin, prodynorphin, and pronociceptin in high vertebrates were employed to evaluate avian neuropeptides and putative peptides together with evolutionary considerations.
